# Parapharyngeal Dissection for Papillary Thyroid Cancer

**DOI:** 10.1089/ve.2018.0141

**Published:** 2019-03-18

**Authors:** Justin Tran, Mark Zafereo

**Affiliations:** Department of Head and Neck Surgery, MD Anderson Cancer Center, Houston, Texas.

**Keywords:** papillary thyroid cancer, parapharyngeal space, lymph node metastases, transcervical, surgery

## Abstract

***Introduction:*** Although lymph node metastases are common with papillary thyroid cancer, parapharyngeal and retropharyngeal lymph node metastases are relatively rare. Although small metastatic parapharyngeal lymph nodes (e.g., <1 cm) may be treated with radioactive iodine or observed, larger lymph nodes may require surgical excision. Surgical approaches to the parapharyngeal and retropharyngeal space include transoral and transcervical.

***Materials and Methods:*** A 47-year-old female presented with a 2 cm conventional papillary thyroid cancer in the right thyroid lobe with central right lateral neck metastases, as well as a 2.5 cm right parapharyngeal lymph node metastasis extending to the skull base. Surgical technique for transcervical resection of the 2.5 cm parapharyngeal lymph node is illustrated, identifying important anatomical structures.

***Results:*** After opening the right neck and removing the right level 2 lymph nodes (not illustrated), the parapharyngeal space is exposed. First, the posterior belly of the digastric and stylohyoid muscles is divided. Next, the hypoglossal nerve is identified and mobilized. Branches of the external carotid artery are then divided and retracted. The sympathetic chain is visualized posterior to the internal carotid artery. The external branch of the superior laryngeal nerve is visualized as it courses posterior to the carotid artery. After gentle retraction of the hypoglossal nerve, superior laryngeal nerve, carotid artery, and sympathetic chain, the parapharyngeal space is exposed with the aforementioned metastatic lymph node. The metastatic lymph node is then freed from the alar fascial and skull base attachments and removed en bloc.

***Conclusion:*** To our knowledge, this is the first video demonstration of a transcervical parapharyngeal lymph node resection in the peer-reviewed literature. Transcervical excision of parapharyngeal and retropharyngeal lymph nodes requires a thorough understanding of the anatomy of the neck and parapharyngeal space, along with a stepwise surgical technique to safely expose the parapharyngeal space.

No competing financial interests exist.

Runtime of video: 8 mins 44 secs

## Graphical abstract

**Figure:**
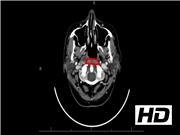

**Figure:**
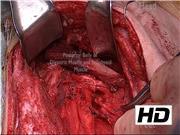

**Figure:**
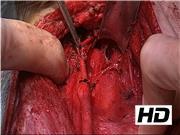

## Video

## Files

**MP4:** 

**PDF:** 

